# Convolutional Neural Network Knowledge Graph Link Prediction Model Based on Relational Memory

**DOI:** 10.1155/2023/3909697

**Published:** 2023-01-31

**Authors:** Ming Shi, Jing Zhao, Donglin Wu

**Affiliations:** School of Computer Science and Technology, Qilu University of Technology (Shandong Academy of Science), Jinan/250353, China

## Abstract

A knowledge graph is a collection of fact triples, a semantic network composed of nodes and edges. Link prediction from knowledge graphs is used to reason about missing parts of triples. Common knowledge graph link prediction models include translation models, semantics matching models, and neural network models. However, the translation models and semantic matching models have relatively simple structures and poor expressiveness. The neural network model can easily ignore the overall structural characteristics of triples and cannot capture the links between entities and relations in low-dimensional space. In response to the above problems, we propose a knowledge graph embedding model based on a relational memory network and convolutional neural network (RMCNN). We encode triple embedding vectors using a relational memory network and decode using a convolutional neural network. First, we will obtain entity and relation vectors by encoding the latent dependencies between entities and relations and some critical information and keeping the translation properties of triples. Then, we compose a matrix of head entity encoding embedding vector, relation encoding embedding vector, and tail entity embedding encoding vector as the input of the convolutional neural network. Finally, we use a convolutional neural network as the decoder and a dimension conversion strategy to improve the information interaction capability of entities and relations in more dimensions. Experiments show that our model achieves significant progress and outperforms existing models and methods on several metrics.

## 1. Introduction

**Figure 1:**
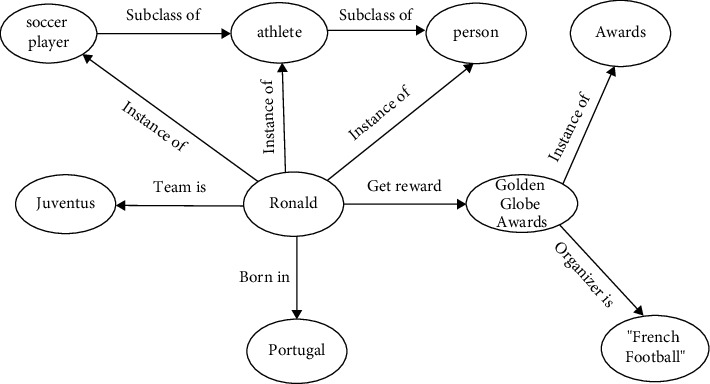
An example of a knowledge graph.

The knowledge graph [1] is a structured semantic knowledge base, which is stored in the form of triples (**h**, **r**, **t**), where **h** is a head entity, **t** is a tail entity, and **r** is the relation between them. Many large knowledge graphs, such as YAGO [2], Freebase [3], and DBpedia [4], use triples to store the entities and relations of the knowledge base. With the advent of the era of artificial intelligence, knowledge graphs have been heavily used, such as critical resources for intelligent applications such as intelligent question answering [5], web search [6], recommender system [7], and sentiment analysis [8, 9].  Figure 1 is an example of a simple knowledge graph.

Although knowledge graphs are widely used, the knowledge graphs are still incomplete; that is, it lacks a large number of effective triples. To make the content of the knowledge graph more complete concept of knowledge graph link prediction is valued by the majority of researchers. An excellent knowledge graph link prediction method is knowledge graph embedding [10]. Knowledge graph embedding aims to learn embedded representations of entities and relations and perform inference and prediction. Typical knowledge graph embedding models include the translation models [11–14] and semantic matching models [15–17], which are easy to train, simple and efficient. However, due to their simple structure, these two models capture fewer features than some deep models, which significantly limits their expressive power. Convolutional neural networks shine in the field of imagery and NLP [18] with their excellent feature extraction capabilities and performance. Recently, researchers have applied CNN to the field of KGE, and some CNN-based models [19–22] have also achieved good results on most datasets. These models generate embedded representations by computing latent connections between entities and relations through convolutional neural networks' powerful nonlinear feature extraction capabilities.

**Figure 2:**
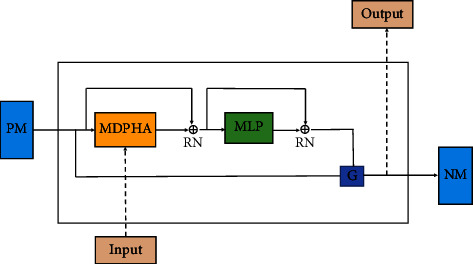
The core of relational memory networks. **PM** stands for previous memory, **NM** for next memory, **MDPHA** for multihead dot product attention, **MLP** for multilayer perceptron, **G** for memory gating, and **RN** for residual network.

Translation models and semantic matching models have relatively simple structures. They only focus on triples' structural information, cannot effectively infer complex semantic connections between entities and relations and perform poorly on datasets with complex relations. Mainstream neural network models cannot capture the connection between entities and relations in low-dimensional space and ignore the translation characteristics between triples. In order to solve the above problems, improve the efficiency of knowledge graph link prediction, increase the fitting ability of the model, and have better performance in dealing with complex relationships, we combine relational memory network and convolutional neural network to enhance the generalization ability of the model. The core of the relational memory network [23] is shown in Figure 2. Specifically, we add positional encoding to the input sequence of head entities, relations, and tail entities. We then use the Transformer self-attention mechanism [24] to interact with the memory matrix to produce encoded vectors. At the same time, in the convolutional decoder part, we propose a dimension conversion strategy, which dramatically increases the feature interaction of entities and relations in more dimensions. Experiments show that our model outperforms the baseline model on most metrics. In summary, the main contributions of this paper are as follows:

- We propose a new knowledge graph embedding model (RMCNN), which uses relational memory networks to encode relations between relations and entities. It can effectively reason about the complex semantic relationships between entities and relations and capture the deep relation between entities and relation embedding vectors.
- We use a dimension conversion strategy on the encoded embedding matrix to increase the number of sliding steps of the convolution kernel and improve the information interaction capabilities of entities and relations in the triple in more dimensions.
- We use four datasets to evaluate the model results by link prediction task. The experiments show that our model has better prediction accuracy than other models.

## 2. Related Work

We introduce the partial translation model in Section 2.1, the semantic matching model in Section 2.2, and the convolutional neural network model in Section 2.3. We compare the entity embedding representation with the relation embedding representation and the scoring function of some models in detail as shown in Table 1.

### 2.1. Translation Models

The TransE [11] model maps the head entity vector, the relation vector, and the tail entity vector to a low-dimensional dense vector space and regards the relation vector as a translation operation from the head entity vector to the tail entity vector. The TransE model has the advantages of fewer parameters and convenient calculation. It performs well on large-scale sparse knowledge graphs. The TransH [12] model defines a hyperplane for each relation. Two entities in the entity space are projected to the hyperplane through the relation mapping matrix. The TransR [13] model defines a relation *r* and the projection matrix *M*_*r*_ of the relation and projects the entity from the entity space to the subspace of the relation *r*. The essence of TransR is to turn the projection vector into a projection matrix, the entity is represented by a vector, and a matrix represents the relation. The TransD [14] model adopts a dual vector design strategy for each entity or relation. Each entity and relation is represented by two vectors (meaning vector and projection vector), one representing its embedding and the other used to construct the projection matrix. The projection matrix used for each entity-relation pair is different, with head and tail entities projected separately. However, the translation model structure is too simplistic to capture the underlying connections between entities and relations.

### 2.2. Semantic Matching Models

RESCAL [15] is the first model to do knowledge graph embedding based on semantic matching, which uses tensor decomposition to build the model. The model represents entities as vectors and relations as matrices and proposes the first scoring function consisting of bilinear products. DistMult [16] improves on RESCAL by restricting its relational matrix to diagonal matrices. ComplEx (Complex Embedding) [17] introduces complex-valued embedding based on DistMult, and the embedding of entities and relations is no longer in the real-valued space but in the complex space. ANALOGY [25] extends RESCAL better to model the reasoning properties of entities and relations. It uses the same bilinear function as RESCAL as the triplet scoring function. RotatE (Rotation Embedding) [26], the main idea is to represent the entity as a complex vector, and the relation is regarded as a rotation from the head entity to the tail entity. However, although the semantic matching model is easy to train, it is straightforward to overfit due to its redundancy, which is a fatal disadvantage for embedding large knowledge graphs.

### 2.3. Convolutional Neural Network Models

The ConvE [19] model is the first model to use CNN to complete the knowledge graph. It reorganizes the head entity vector and the relation vector and combines them into a matrix as the input of the convolutional layer of CNN. ConvE uses different convolution kernels for convolution and outputs feature maps. It maps these feature maps to a vector and uses that vector to do a dot product with the tail entity to get the triple score. 1D convolution can only capture the interaction at the splicing of vectors. ConvE uses 2D convolution in the image domain to obtain more interactions than 1D convolution. However, 2D convolution can only capture part of the interaction, so the interaction between entities and relations is still insufficient. Therefore, to maximize the interaction between entities and relations, the researchers proposed the ConvR [21] model, which uses the embedding of the relation as a convolution filter and performs convolution operations on the embedding of the head entity, which can fully interact between the entity and the relation. the InteractE model focuses on how to increase the interaction between entities and relations. InteractE [27] mainly increases the interaction between entities and relations through feature replacement, rashape operations, and circular convolution. JointE [28] combines 1D and 2D convolutions to embed the knowledge map, where 1D convolution is used to obtain explicit knowledge and 2D convolution is used to obtain deep knowledge. However, these convolutional neural network models ignore triples' translation properties and do not pay attention to the global features of triples.

## 3. Methods

This section introduces the symbols we use and their definitions in Section 3.1, our model framework in Section 3.2, and the loss function we use in Section 3.3.

### 3.1. Definition

The knowledge graph **G**_**r**_ is a set of valid triples in the form of (head entity, relation, tail entity) expressed as (**h**, **r**, **t**). Among them, *h*, *t* ∈ **E** and r ∈ **R**, where **E** is the set of entities and **R** is the set of relations. We define **v**_**h**_, **v**_**r**_, **v**_**t**_ ∈ *ℝ*^*D*^ to represent the embedding representation of the head entity, the relation, and the tail entity, respectively. We define *f*(*h*, *r*, *t*) as the scoring function. If the triple is valid, the corresponding score will be higher.

### 3.2. The Framework of the Proposed Model

**Figure 3:**
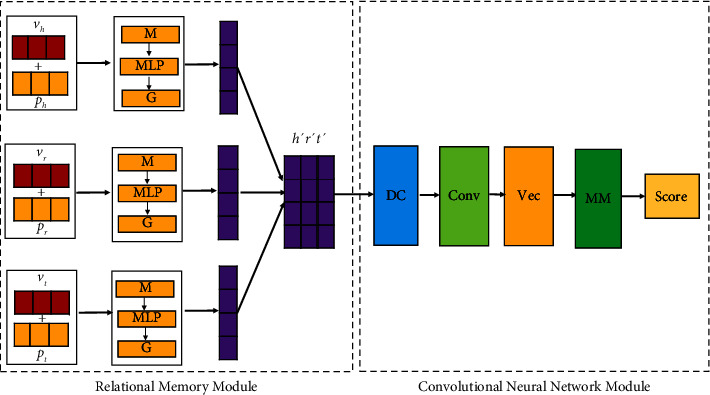
The detailed structure diagram of our model. **p** stands for position code, (*M*) stands for memory, **MLP** for multilayer perceptron, **G** for memory gating, **DC** stands for dimension conversion, **Conv** for convolution operation, **Vec** for vectorization operation, and **MM** stands for matrix multiplication.

The model structure of this paper is shown in Figure 3, mainly consists of two parts: the relational memory module and the convolutional neural network module. The relational memory module, which is composed of multilayer perceptrons and memory gates, encodes the potential dependencies and important parts of the information between entities and relations and forms a coded embedding vector. The convolutional neural network module needs to go through five processes, dimensional conversion, convolution operation, feature map vectorization, linear mapping, and dot product operation.

We believe that the relative positions of the head entity, relation, and tail entity are of great significance for reasoning about fact triples. Therefore, we add the corresponding position embedding codes to the head entity vector, relation vector, and tail entity vector. Given a triple (**h**, **r**, **t**), the vector representation of **x**_**h**_, **x**_**r**_, **x**_**t**_ can be obtained as shown in the following equations:

(1) $$\begin{array}{c} \begin{array}{c} x_{h}=W\left(v_{h}+p_{h}\right)+b_{1} \end{array}\text{,} \end{array}$$

(2) $$\begin{array}{c} \begin{array}{c} x_{r}=W\left(v_{r}+p_{r}\right)+b_{2} \end{array}\text{,} \end{array}$$

(3) $$\begin{array}{c} x_{t}=W\left(v_{t}+p_{t}\right)+b_{3}\text{,} \end{array}$$

where *p*_*h*_, *p*_*r*_,*p*_*t*_ ∈ *ℝ*^*D*^ represent the position encoding embedding vector of head entity, relation, and tail entity, **W** ∈ *ℝ*^*N*×*D*^ is a projection weight matrix. Position coding is used to determine the potential semantic connection of entities and relations in the low-dimensional representation space. *D* represents the embedding dimension of entities and relations, *N* stands the size of memory.

In this paper, the memory matrix is defined as *M* ∈ *ℝ*^U×N^ consisting of *U* rows and *N* columns, where each row represents a memory slot. In our research, we use *M*^(*e*)^ to represent the memory matrix at time *e*, and *M*_*i*_^*e*^ ∈ *ℝ*^*N*^ to represent the i-th memory slot at time *e*. The attention mechanism in Transformer uses the multihead attention mechanism to update the vector to make the input vector interact with the memory matrix. We use **x**^(**e**)^ to update *M*_*i*_^*e*^ according to the proposal made by the relational memory network, and effectively capture the potential dependencies between triples, where ${\hat{M}}_{i}^{e+1}$ and ${\hat{M}}_{i}^{e+1,c}$ is shown by the following equations:

(4) $$\begin{array}{c} \begin{array}{c} {\hat{M}}_{i}^{\left(e+1\right)}=\left[{\hat{M}}_{i}^{\left(e+1\right),1}⊕{\hat{M}}_{i}^{\left(e+1\right),1}⊕\ldots ⊕{\hat{M}}_{i}^{\left(e+1\right),C}\right] \end{array}\text{,} \end{array}$$

(5) $$\begin{array}{c} \begin{array}{c} {\hat{M}}_{i}^{\left(e+1\right),c}=\alpha_{i,U+1,c}\left(W^{c,V}x^{\left(e\right)}\right)+\overset{U}{\underset{j=1}{\sum }}\alpha_{i,j,c}\left(W^{c,V}M_{j}^{\left(e\right)}\right) \end{array}\text{,} \end{array}$$

where ${\hat{M}}_{i}^{e+1}$ represents the i-th memory slot at the *e* + 1-th time, ${\hat{M}}_{i}^{e+1,c}$ represents *c*-th head of the multihead attention mechanism, *C* is the number of heads in the multihead attention mechanism, and ⊕ represents the splicing operation, which stitches the results of each head of the multihead attention mechanism. **W**^**c**,**V**^∈*ℝ*^*n*×*N*^ is a value projection matrix, in which *n* is the head size and *N*=*nC*, *α* is the weight value of the attention mechanism calculated by the softmax function, *β* is the scalar value obtained by the dot product of the query matrix and the key matrix, as shown in the following equations:

(6) $$\begin{array}{c} \text{,} \end{array}$$

(7) $$\begin{array}{c} \alpha_{i,U+1,c}=\frac{\exp\left(\beta_{i,U+1,c}\right)}{\overset{U+1}{\underset{m=1}{\sum }}\exp\left(\beta_{i,m,c}\right)}\text{,} \end{array}$$

(8) $$\begin{array}{c} \beta_{i,j,c}=\frac{{\left(W^{c,Q}M_{i}^{\left(e\right)}\right)}^{T}\left(W^{c,K}M_{j}^{\left(e\right)}\right)}{\sqrt{n}}\text{,} \end{array}$$

(9) $$\begin{array}{c} \beta_{i,U+1,c}=\frac{{\left(W^{c,Q}M_{i}^{\left(e\right)}\right)}^{T}\left(W^{c,K}x^{\left(e\right)}\right)}{\sqrt{n}}\text{,} \end{array}$$

where **W**^**c**,**Q**^ ∈ *ℝ*^*n*×*N*^ and **W**^**c**,**K**^ ∈ *ℝ*^*n*×*N*^ are the query projection matrix and the key projection matrix. In this paper, the residual network is connected between **x**^(*e*)^ and ${\hat{M}}_{i}^{e+1}$ to ensure its good performance, and the results of the residual network are fed to the multilayer perceptron and memory gating. Then, it generates N-dimensional encoded embedding vectors **h**′, **r**′, **t**′ ∈ *ℝ*^*N*^ for time *e* and the next memory slots ${\hat{M}}_{i}^{e+1}$ for time *e*+1.

**Figure 4:**
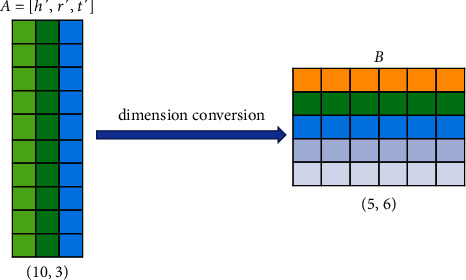
Our dimension conversion strategy. The figure is to change the matrix of 10 rows and 3 columns into 5 rows and 6 columns.

As a result, we obtain a sequence of 3 encoded vectors (**h**′, **r**′, **t**′) for the triple (**h**, **r**, **t**). We use a convolutional neural network and a matrix **A**=[**h**′, **r**′, **t**′] ∈ *ℝ*^*N*×3^ of encoded embedding vectors output by the relational memory network as the input of the convolutional neural network. RMCNN performs a dimension conversion strategy on **A** matrix. Specifically, assuming that the vector dimension of each element in the triple is 100, using a convolution kernel of shape 3 × 3 will slide 98 times on the triple matrix of shape 100 × 3. The RMCNN model adopts a dimension conversion strategy, which can convert a 100 × 3 triple matrix into a 10 × 30 shape. Assuming that 3 × 3 convolution kernels are also used, the number of times each convolution kernel slides on the convolution kernel is 224, and the number of sliding times of the convolution kernel on the triple matrix increases significantly. Due to the triple matrix dimension conversion strategy, our model improves the information interaction ability of entities and relations in the triple matrix in more dimensions. Our specific dimension conversion strategy is shown in the following Figure 4.

The RMCNN model performs a dimension conversion strategy on the **A** matrix to obtain the **B** matrix, **B** ∈ *ℝ*^*m*×*s*^, *m* × *s*=*k* × 3. We use different 2D convolution kernels *ω* to convolve the matrix **B** to extract the features. |Ω| is used to represent the set of convolution filters *ω*, *τ*=|Ω| represents the number of convolution kernels. And, it is assumed that the dimension of the feature maps obtained by the convolution operation is *d*_1_ × *d*_2_. Our model combines these feature matrices and reshapes them into a vector *d*_*vec*_ ∈ *ℝ*^*d*_1_*d*_2_*τ*×1^. The vector *d*_*vec*_ is first multiplied by the weight matrix **W** ∈ *ℝ*^*u*×*d*_1_*d*_2_*τ*^ and mapped into the u-dimensional vector space, and then the dot product operation is performed with the weight vector **w** ∈ *ℝ*^*u*×1^ to obtain the score of the triple. Therefore, our scoring function is defined as shown in the following equation:

(10) $$\begin{array}{c} \begin{array}{c} f\left(h,r,t\right)=vec\left(g\left(B*\;Ω\right)\right)\times W\cdot w \end{array}\text{,} \end{array}$$

where ^*∗*^ represents convolution operation, × represents the multiplication operation of the matrix, · represents the dot product operation between vectors, *vec* represents the vectorization operation of the combined characteristic matrix, *g* represents the activation function, Ω represents the set of convolution kernels, **W** is the projection weight matrix, and **w** is the weight vector.

### 3.3. Loss Function

After we get the scoring function for the triples, the RMCNN model can calculate the score for each triple. Usually, vaild triples will get higher scores than invalid triples. The nonconvex relaxations usually achieve better performance than the convex case since the former can achieve a nearly unbiased solver [29–31]. Therefore, we choose the log logistic regression function as our loss function. Furthermore, we employ the Adam optimizer to train our model by minimizing the following loss function:

(11) $$\begin{array}{c} L=\underset{\left(h,r,t\right)\in \left\{G_{r}\cup {G_{r}}^{\prime }\right\}}{\sum }\log\;\left(1+\exp\;\left(-\delta_{\left(h,r,t\right)}\cdot f\left(h,r,t\right)\right)\right)\text{,}\\ \delta_{\left(h,r,t\right)}=\left\{\begin{array}{c} 1\;\left(h,r,t\right)\in G_{r}\\ -1\;\left(h,r,t\right)\in {G_{r}}^{\prime } \end{array}\right. \end{array}$$

where *G*_*r*_ and *G*_*r*_′ are the sets of valid and invalid triples, respectively. *G*_*r*_ is generated by destroying valid triples in *G*_*r*_′.

## 4. Experiment

In this section, we evaluate the performance of RMCNN. The experimental results show that our model has a good improvement in performance compared with the previous models. We use classic link prediction experiments to validate our model. In Section 4.1, we introduce the dataset used; in Section 4.2, we illustrate the hyperparameters used; in Section 4.3, we clarify our experimental metrics; in Section 4.4, we perform the empirical analysis; in Section 4.5, we conduct ablation experiments.

### 4.1. Datasets

We execute many experiments on link prediction tasks on the following benchmark datasets: YAGO3-10 [2], Kinship [32], FB15k-237 [27], and WN18RR [19]. The details of these datasets are shown in Table 2. Since there are many reversible relations in FB15k and WN18, it is easier to predict most triples, so we adopt FB15k-237 and WN18RR with the reversible relation removed. Kinship is a small dataset with kinship relations. YAGO3-10 is the largest of the four datasets and it is a subset of YAGO3.

### 4.2. Hyperparameters

In our experiments, we acquired the best accuracy on the validation set when using a single memory slot (i.e., *U* = 1). This paper sets the following: the number of heads in the multihead attention mechanism *C*={1,2,3,4,5}, the size of the head in the multihead attention mechanism *n*={128,256,512,1024}, the number of layers of the multilayer perceptron *l*={1,2,3,4}, the number of convolution filter |Ω|={256,512,1024}, the memory matrix size *N*=*nC*. To maximize the learning effect of our model learning parameters, we use Adam's initial learning rate *γ*. The specific hyperparameters we use are shown in Table 3.

### 4.3. Evaluation Metrics

Link prediction predicts the relation between entities and entities that are missing triples in the knowledge graph. For example, given a triple (Ronald, born_in, ?), where the head entity is Ronald, the relation is born_in, and the tail entity is missing, completing the triple, add Portugal to the triple.

In this study, we use standard metrics to evaluate our model, similar to previous work: mean reciprocal rank (**MRR**) and percentage of entering top *k* (**Hit@k**). **MRR** is the average of the reciprocal scores of predicted correct samples in all test samples. **Hits@k** refers to the proportion that the score of the predicted correct sample is higher than the *k*-th or equal to the *k*-th among all test samples. Given triples (*h*, *r*, *t*) in the test set, we use a scoring function to score them and randomly generated negative triples and sort their scores in descending order. The specific calculation steps are shown in the following equations:

(12) $$\begin{array}{c} MRR=\frac{1}{\left|NS\right|}\overset{\left|NS\right|}{\underset{i=1}{\sum }}\frac{1}{{rank}_{i}}\text{,} \end{array}$$

(13) $$\begin{array}{c} Hit@k=\frac{1}{\left|NS\right|}\overset{\left|NS\right|}{\underset{i=1}{\sum }}Indi\left({rank}_{i}\leq k\right), \end{array}$$

where |*NS*| denotes the number of triples and rank_*i*_ denotes the link prediction rank of the *i* − *th* triple. Indi() is an indicator function (if the condition is true, the function value is 1. Otherwise, it is 0), and the value of *k* generally takes 1, 3, or 10.

### 4.4. Analysis of Results

We demonstrate the performance of different models on four benchmark datasets and give further analysis. The results of our specific link predictions are shown in Tables 4 and 5, where the highest score is shown in bold and the second highest score is underlined. However, the semantic matching model is prone to overfitting, causing its performance to lag behind the convolutional neural network model. MRR is the ability of our model to correctly represent triple relations. The improvement in this metric indicates that our model is able to learn triple vectors well. On the WN18RR dataset, compared with ConvE, our model has a good improvement in various metrics, with MRR increasing by 10% and Hit@10 increasing by 3.8%. Compared with the best baseline model InteractE, MRR is improved by 1.2%, and Hit@10 is improved by 2.1%. On the FB15k-237 dataset, compared with InteractE, which also uses a convolutional neural network, RMCNN improves MRR by 1.4% and Hit@3 by 1.1%. InteractE also shows excellent performance on the FB15k-237 dataset with many relations and few entities. Even compared with the latest JoinE, our model has good advantages in two datasets.

In addition, we also adopt a large dataset YAGO3-10 and a smaller dataset, Kinship, to evaluate our model. We use two classic semantic matching models, DisMult, and ComplEx, and three typical convolutional neural network models, ConvE, HypER, and InteractE, as our baseline models. After experiments, our results are shown in Table 4. On the YAGO3-10 dataset, our model outperforms other models on all metrics, compared with InteractE, RMCNN achieves 1.5%, 1.9%, 2%, and 2.3% improvement on MRR, Hit@10, Hit@3, and Hit@1, respectively. We found that models based on convolutional neural networks outperformed semantic matching models due to the nonlinear nature of convolutional neural networks. To better verify the performance of our model, we also conduct experiments on a small dataset, Kinship. After comparison, our model performance far outperforms other baseline models. After comparison, our model performance far outperforms other baseline models. This also shows that our model can perform excellent modeling of knowledge graphs, whether it is a large dataset or a small dataset.

After the experimental results of the above four datasets, we can see that our model has surpassed the KGE models ConvE, InteractE, and JointE, which are also based on convolutional neural networks, in many metrics and have shown in various datasets. The excellent performance reflects the good robustness of our model.

### 4.5. Ablation Experiments

We adopt ablation experiments in order to prove the effectiveness of the relational memory network and dimension conversion strategy. Tables 6 and 7 show the results of our ablation experiments. RMCNN (**RM**) uses only a relational memory network; RMCNN (**DC**) uses only a dimensional conversion strategy. RMCNN (**RM**) achieves excellent performance using only the relational memory network, showing that the relational memory network can encode and remember latent dependencies between entities and relations well. The performance of RMCNN cannot be fully achieved using only the relational memory network, where MRR drops from 0.358 to 0.349 on FB15k-237, 0.473 to 0.463 on WN18RR, 0.557 to 0.521 on YAGO3-10, 0.872 to 0.854 on Kinship; Hit@10 drops from 0.535 to 0.529 on FB15k-237, and drop from 0.540 to 0.531 on WN18RR. Scrutinizing these changes, we can verify that our dimensional transformation strategy improves the interaction between entities and relations in more dimensions.

In conclusion, the results of our ablation experiments demonstrate that high performance can be achieved using only relational memory networks. However, its link prediction performance is still inferior to our RMCNN model. These experimental analyses demonstrate that the relational memory network encoding entity and relation embeddings significantly contribute significantly to the link prediction task. In contrast, the dimension conversion strategy that captures the interactions of entities and relations in more dimensions plays an auxiliary role. Therefore, only by combining the two can we fully grasp the potential links between entities and relations, improve the interaction between entities and relations, and obtain better link prediction capabilities.

## 5. Conclusion

This paper proposes a model based on relational memory networks and convolutional neural networks. The model uses the relational memory network to encode triples and uses the convolutional neural network to decode, which improves the efficiency of knowledge graph link prediction. Firstly, the relational memory network is used to encode the entity and relation vector, so as to fully retain the important information of entities and relations. Then, in the convolutional neural network decoding part, we use a dimensional conversion strategy to add interactions between entities and relations in more dimensions. A limitation of the current work is that the proposed neural network structure needs to be designed manually. In future work, we will consider using neural network architecture search methods to search for optimal convolutional neural network structures for a specific data set, which will be a worthwhile direction to explore.

## Figures and Tables

**Table 1:** Summary of link prediction model of knowledge graph.

Models	Entity embedding	Relation embedding	Score function
TransE	**v**_**h**_, **v**_**t**_ ∈ *ℝ*^*D*^	**v**_**r**_ ∈ *ℝ*^*D*^	−||**v**_**h**_+**v**_**r**_ − **v**_**t**_||_1/2_
TransR	**v**_**h**_, **v**_**t**_ ∈ *ℝ*^*D*^	**v**_**r**_ ∈ *ℝ*^*K*^, **M**_**r**_ ∈ *ℝ*^*K*×*D*^	−||**M**_**r**_**v**_**h**_+**v**_**r**_ − **M**_**r**_**v**_**t**_||_2_^2^
RESCAL	**v**_**h**_, **v**_**t**_ ∈ *ℝ*^*D*^	**M**_**r**_ ∈ *ℝ*^*D*×*D*^	**v**_**h**_^*T*^ **M**_**r**_ **v**_**t**_
DistMult	**v**_**h**_, **v**_**t**_ ∈ *ℝ*^*D*^	**v**_**r**_ ∈ *ℝ*^*D*^	**v**_**h**_^*T*^ **d** **i** **ag**(**M**_**r**_)**v**_**t**_
ComplEx	**v**_**h**_, **v**_**t**_ ∈ *ℂ*^*D*^	**v**_**r**_ ∈ *ℂ*^*D*^	*Re*[**v**_**h**_^*T*^**di** **ag**(**M**)_**r**_)**v**_**t**_]
RotatE	**v**_**h**_, **v**_**t**_ ∈ *ℂ*^*D*^	**v**_**r**_ ∈ *ℂ*^*D*^	−||**v**_**h**_⊙**v**_**r**_ − **v**_**t**_||
ConvE	**M**_**h**_ ∈ *ℝ*^*D*_1_×*D*_2_^, **v**_**t**_ ∈ *ℝ*^*D*^	**M**_**r**_ ∈ *ℝ*^*D*_1_×*D*_2_^	*σ*(vec(*σ*([**M**_**h**_; **M**_**r**_]*∗ω*))**W**)**v**_**t**_
ConvR	**v**_**h**_, **v**_**t**_ ∈ *ℝ*^*D*^	*ω*_*r*_ ∈ *ℝ*^*m*×*n*^	*g*(*Wg*([**v**_**h**_]*∗ω*_*r*_)+*b*)**v**_**t**_
HypER	**v**_**h**_, **v**_**t**_ ∈ *ℝ*^*D*^	*ω*_*r*_ ∈ *ℝ*^*D*_*r*_^	*σ*(vec(**v**_**h**_*∗vec*^−1^(*ω*_*r*_**H**)))**W**)**v**_**t**_]
InteractE	**M**_**h**_ ∈ *ℝ*^*D*_1_×*D*_2_^, **v**_**t**_ ∈ *ℝ*^*D*^	**M**_**r**_ ∈ *ℝ*^*D*_1_×*D*_2_^	*σ*(vec(*σ*([**M**_**h**_; **M**_**r**_]*∗∗ω*))**W**)**v**_**t**_
RMCNN	**v**_**h**_, **v**_**t**_ ∈ *ℝ*^*D*^	**v**_**r**_ ∈ *ℝ*^*D*^	vec(*g*(**B***∗* Ω)) × **W***· ***w**

**Table 2:** Datasets.

Datasets	Entities	Relations	Train	Vaild	Test
FB15k-237	14541	237	272115	17535	20466
WN18RR	10943	11	86835	3034	3134
YAGO3-10	123182	37	1079040	5000	5000
Kinship	104	25	8544	1068	1074

**Table 3:** Hyperparameters.

Datasets	*C*	*n*	*l*	|Ω|	*γ*
FB15k-237	2	1024	4	512	0.03
WN18RR	4	512	4	256	0.03
YAGO3-10	4	1024	4	1024	0.03
Kinship	3	1024	4	256	0.03

**Table 4:** Results of the link prediction on WN18RR and FB15k-237 datasets.

Models	WN18RR				FB15k-237			
	MRR	Hit@10	Hit@3	Hit@1	MRR	Hit@10	Hit@3	Hit@1
TransE [11]	0.182	0.444	0.295	0.027	0.257	0.420	0.284	0.174
DisMult [16]	0.430	0.490	0.440	0.390	0.241	0.419	0.263	0.155
ComplEx [17]	0.440	0.410	0.460	0.353	0.249	0.428	0.275	0.158
ConvE [19]	0.430	0.520	0.440	0.400	0.325	0.501	0.356	0.237
HypER [33]	0.468	0.526	0.482	*0.438*	0.336	0.514	0.367	0.248
InteractE [27]	0.467	0.529	*0.482*	0.435	0.353	*0.541*	0.390	0.260
JointE [28]	*0.471*	0.537	**0.483**	*0.438*	*0.356*	**0.543**	*0.393*	**0.262**
RMCNN	**0.473**	**0.540**	0.479	**0.440**	**0.358**	0.535	**0.394**	*0.255*

**Table 5:** Results of the link prediction on YAGO3-10 and kinship.

Models	YAGO3-10				Kinship			
	MRR	Hit@10	Hit@3	Hit@1	MRR	Hit@10	Hit@3	Hit@1
TransE [11]	0.501	0.673	0.392	0.405	0.309	0.809	0.643	*0.841*
DisMult [16]	0.340	0.540	0.380	0.240	0.685	0.943	0.766	0.553
ComplEx [17]	0.360	0.550	0.400	0.560	0.861	0.977	*0.935*	0.780
ConvE [19]	0.440	0.620	0.490	0.350	0.830	*0.980*	0.920	0.740
HypER [33]	0.533	0.678	0.580	0.455	**0.879**	0.810	**0.942**	**0.986**
InteractE [27]	*0.549*	*0.685*	*0.595*	*0.472*	0.867	0.792	0.932	0.664
RMCNN	**0.557**	**0.698**	**0.607**	**0.483**	*0.872*	**0.984**	**0.942**	0.803

**Table 6:** Ablation experiments on FB15K-237 and WN18RR.

Model	FB15k-237				WN18RR			
	MRR	Hit@10	Hit@3	Hit@1	MRR	Hit@10	Hit@3	Hit@1
RMCNN **(RM)**	0.349	0.529	0.385	0.248	0.467	0.531	0.472	0.432
RMCNN **(DC)**	0.331	0.518	0.371	0.236	0.456	0.492	0.465	0.428
RMCNN	0.358	0.535	0.394	0.255	0.473	0.540	0.479	0.440

**Table 7:** Ablation experiments on YAGO3-10 and Kinship.

Model	YAGO3-10				Kinship			
	MRR	Hit@10	Hit@3	Hit@1	MRR	Hit@10	Hit@3	Hit@1
RMCNN **(RM)**	0.521	0.654	0.584	0.396	0.854	0.942	0.933	0.765
RMCNN **(DC)**	0.545	0.598	0.577	0.461	0.866	0.958	0.921	0.786
RMCNN	0.557	0.698	0.607	0.483	0.872	0.984	0.942	0.803

## Data Availability

The labeled data set used to support the findings of this study is available from the author upon request.
